# What are gender differences in lower limb muscle activity during jump–landing tasks? A systematic review and meta-analysis

**DOI:** 10.1186/s13102-022-00469-3

**Published:** 2022-04-28

**Authors:** Mohammad Seyedahmadi, Hooman Minoonejad, Mohammad Karimizadeh Ardakani, Zahra Heidari, Mohammad Bayattork, Hadi Akbari

**Affiliations:** 1 Department of Sport Sciences, Velayat University, Iranshahr, Iran; 2 Department of Health and Sport Medicine, University of Tehran, Tehran, Iran; 3 Department of Epidemiology and Biostatistics, Isfahan University of Medical Sciences, Isfahan, Iran; 4 Department of Sport Sciences, University of Hormozgan, Bandar Abbas, Iran; 5 Department of Sport Sciences, University of Zabol, Zabol, Iran

**Keywords:** Electromyography, Muscle activity, Jump–landing, Anterior cruciate ligament, Gender

## Abstract

**Background:**

Gender differences in muscle activity during landing have been proposed as a possible contributing factor to the greater incidence of anterior cruciate ligament injuries in women. Conflicting results among a few studies in this regard makes it impossible to reach correct conclusions.

**Objectives:**

The aim of this study was systematic review and the meta-analysis of previous studies which have compared the electromyographic activity of lower limb muscles in gluteus muscles (maximus and medius), quadriceps (rectus femoris, vastus medialis and lateralis), hamstrings (biceps femoris and semimembranosus), and gastrocnemius in men and women in jump–landing task.

**Methods:**

A systematic search of the PubMed, SCOPUS, Science Direct databases was performed for eligible articles in October 2020. Cross-sectional studies that compared the muscle activity of male and female athletes without a history of previous injury in the jump–landing task were included. Unisex and non-athlete's studies were extracted from the included studies. The data were synthesized using a fixed and random effects model.

**Results:**

Eight studies involving 145 participants were included. All participants were people who participated in regular exercises. The meta-analysis of timing and muscle activity was performed in the feedforward (pre contact) and feedback (post contact) stages. There were no significant differences in the muscle activity of biceps femoris (MD = −12.01; 95% CI − 51.49 to 27.47; p = 0.55; I^2^ = 87%), vastus medialis (MD = −53.46; 95% CI − 129.73 to 22.81; p = 0.17; I^2^ = 91%), semimembranosus (MD = 1.81; 95% CI − 6.44 to 10.07; p = 0.67; I^2^ = 0%), gluteus medius (MD = −3.14; 95% CI − 14.24 to 7.96; p = 0.58; I^2^ = 48%), and rectus femoris (MD = −5.83; 95% CI − 14.57 to 2.92; p = 0.19; I^2^ = 87%) in the pre contact phase between two sexes. There was a significant difference between men and women in the activity of vastus lateralis muscle in the post contact phase (MD = −34.90; 95% CI − 48.23 to − 21.57). No significant difference was observed between the men and women in the timing of semimembranosus (MD = 23.53; 95% CI − 14.49 to 61.54; p = 0.23; I^2^ = 56%) and biceps femoris muscle activity (MD = −46.84; 95% CI − 97.50 to 3.83; p = 0.07; I^2^ = 82%).

**Conclusion:**

The results showed that in all lower limb muscles except vastus lateralis there were no significant differences between muscle activity and muscle contraction timing in both sexes before and after foot contact. Therefore, it can be concluded that the reason for the greater susceptibility of ACL injuries in women than men is maybe related to other factors such as biomechanical and hormonal. Additional good quality research in this regard is required to strengthen these conclusions.

## Background

Anterior cruciate ligament (ACL) injuries are common in dynamic sports activities in athletes (16–39 years old), and account for about 26% of all injuries to the knee joint [1, 2]. The cost of treating and rehabilitating each ACL injury is approximately $17,000 [3]. More than 25% of people whose ACL are injured cannot return to their previous levels of activity even after successful surgery and rehabilitation [4]. ACL injuries have caused a great deal of concern in sport due to the severe consequences such as losing exercise time, negative performance effects, early onset of knee osteoarthritis, and decreased knee function [5, 6]. The risk of ACL injury is 2 to 8 times greater in women than men [7, 8]. The most common mechanism of ACL injury is non-contact and one of the most common activities that may lead to the ACL injury is jump–landing [9]. So, improper technique during jump–landing manoeuvres can cause considerable force on the ACL and rupture it [10–13]. In order to create an intervention to prevent ACL injuries, sufficient understanding of its mechanisms and risk factors is essential.

Although sport movements (sudden decrease and increase of acceleration, rotating, cutting, pivoting and jump–landing) lead to excessive loads on the knee in both sexes, these manoeuvres are more risky in women than men [14]. Higher incidence of injuries in women has led to extensive studies on gender differences [15, 16]. There are three main reasons for women's susceptibility to an ACL injury, including anatomical (lower extremity alignment, posterior tibial slope, notch parameters etc.), hormonal (ACL injury more common in pre-ovulatory phase due to high oestrogen), and neuromuscular factors (differences in movements and muscle activation patterns) [17, 18]. Contrary to anatomical risk factors which are not modifiable without surgery, neuromuscular deficits are modifiable [18]. So, the focus must be on modifiable risk factors in order to mitigate the risk of ACL injury [18].

Studies examining the effects of muscle activation patterns and neuromuscular factors on women's ACL injuries indicated that women have deficiencies in the neuromuscular control system compared to men [19–21]. The activity of different muscle groups can increase or decrease the strain on the knee ligaments [22–24]. The level of balanced activity of the agonist and antagonist muscles of the knee and hip to stabilize the joints indicates the sensory-motor importance of those muscles. The hip and knee muscles must act in a perfect balance, at the right time, at the right duration and with the right combination of forces [21]. Therefore, the improper loading, direction, and function of the muscles around the knee can predispose the ACL to injury.

It has been shown that the timing of quadriceps, hamstrings, and gastrocnemius muscles is associated with ACL injury [25]. How and when these muscles are activated affects the knee's ability to optimize knee stiffness, absorb and dissipate forces, thereby preventing ACL injury [25]. The ratio of strength and activation time of the hamstring to the quadriceps has also been introduced as an essential factor in estimating the ACL injury [26]. A recent study has shown that the calf muscles are also involved in activities that put the ACL at risk for ruptures in cutting movements and jump–landing tasks [27], so that the gastrocnemius muscle exerts loads on ACL in closed kinetic chain activities and has an antagonistic role for ACL [28–30]. It seems that by examining the difference in muscle activity in male and female athletes and confirming this difference, different training programs can be designed to prevent ACL injury. The results of previous research in this regard have been contradictory [31–34], so that a systematic review and meta-analysis is required to correct conclusions about the gender differences in the timing and muscle activity of the lower limb muscles in the jump–landing task. Therefore, the aim of this systematic review and meta-analysis was to compare the electromyographic activity of lower limb muscles in gluteus muscles (maximus and medius), quadriceps (rectus femoris, vastus medialis and lateralis), hamstrings (biceps femoris and semimembranosus), and gastrocnemius in men and women in jumping-landing task.

## Materials and methods

### Protocol and registration

This systematic review was completed according to the Preferred Reporting Items for Systematic Reviews and Meta-Analyses (PRISMA) guidelines [35]. It was also registered in the PROSPERO database on 07/02/2021 (registration number: CRD 42021229881).

### Eligibility criteria

A study was included in this systematic review if it met the following criteria: a) Type of study: cross-sectional studies; b) Type of participants: gender comparison studies in which samples were male and female athletes with no history of sport injury; c) the competitional levels: recreational or university athletes; d) Type of measurement: lower extremity electromyography activity was measured before or after ground contact [36], and lower extremity electromyography activity was determined by a well-defined detection technique. d) The articles were available in English; e) The studies were selected for statistical analysis as meta-analysis if the variables of mean values and standard deviation of muscle activity in the feedback or feedforward stage of the target muscles as well as the number of subjects were also reported in the studies. Studies were excluded if: a) their samples were non-athletes; b) they were unisex studies; c) they were qualitative studies, survey studies, and experimental studies that prescribed a training course; d) the tasks used in them were hopping, stop jump, and cutting.

### Search strategy

Articles in English published until 31 October, 2020 were searched in the PubMed, Scopus, and Science Direct databases. An updated search was performed in August 2021, which yielded no additional results. The search keywords were "electromyography OR muscle activity OR EMG", "jump–landing", "Anterior Cruciate Ligament" OR "ACL", and "gender". A hand search of reference lists was also performed.

### Risk of bias

Two of the authors (MSA and HA) assessed the included studies for bias using the Cochrane Collaboration’s risk of bias tool [37]. Publication bias was assessed by funnel plot analysis generated by Review Manager Version 5.4 (The Cochrane Collaboration, Denmark).

### Data collection process

Two authors (MSA and HA) independently conducted a systematic search to identify the relevant titles and abstracts from the databases. The search results were entered into the EndNote (version X9 for windows) and duplicates from various databases were deleted. Both authors reviewed the titles and abstracts for eligibility before viewing the full texts. Also, reference lists of eligible studies were selected for further eligible studies. Both authors (MSA and HA) independently reviewed full-text studies and compared the studies based on inclusion and exclusion criteria.

The Excluded articles were discussed by two authors (MSA and HA) and whenever the two authors disagreed about an article the issue was resolved by other authors (HM, MKZ, MBT), and then final decision was made. The authors classified and sorted the results of the studies according to the form of EMG data report before and after ground contact, registered EMG variables (onset/amplitude), and the comparison of the participants. In addition, the following muscle activity ' average values for onset were registered and the related SD or SE were extracted: Rectus Femoris (RF) Vastus Lateralis (VL) and Vastus Medialis (VM), Biceps Femoris (BF), Semimembranosus (SM), Medial Gastrocnemius (MG), Lateral Gastrocnemius (LG), Gluteus Medius (GMed) and Gluteus Maximus (GMax). The Semitendinosus muscle (ST) or the SM muscle was used to collect EMG data for the medial hamstrings.

### Quality evaluation

A methodological quality assessment consisting of an adapted version of the Quality Index was developed by Downs and Black [36, 38]. To evaluate the quality of the selected studies, a modified checklist was used which was taken from Downs and Black's checklist [38]. Electromyographic activity during jump–landing for different muscles was reviewed in 8 articles [31–34, 39–43]. The results of the quality evaluation were given in Table 1. A score of 1 was allocated for each question where the answer was ‘‘yes’’ and a score of 0 was allocated for each question where the answer was ‘‘no’’. The number of questions in the modified questionnaire was 9 questions. All studies scored highly (≥ 7) for the adapted Quality Index and no serious deficiencies in any area were identified.

**Table 1:** Quality evaluation

References	Reporting					External validity	Internal validity (bias)		Power	
	Q 1	Q 2	Q 3	Q 4	Q 6	Q 11	Q 18	Q 20	Q 27	Score
Rozzi et al. [34]	Yes	Yes	Yes	Yes	Yes	No	Yes	Yes	No	7
Garrison et al. [40]	Yes	Yes	Yes	Yes	Yes	No	Yes	Yes	No	7
Zazulak et al. [42]	Yes	Yes	Yes	Yes	Yes	No	Yes	Yes	No	7
Carcia and Martin [39]	Yes	Yes	Yes	Yes	Yes	No	Yes	Yes	No	7
Nagano et al. [41]	Yes	Yes	Yes	Yes	Yes	No	Yes	Yes	No	7
Ebben et al. [32]	Yes	Yes	Yes	Yes	Yes	Yes	Yes	Yes	No	8
de Britto et al. [31]	Yes	Yes	Yes	Yes	Yes	No	Yes	Yes	No	7
Ogasawara et al. [33]	Yes	Yes	Yes	Yes	Yes	No	Yes	Yes	No	7

Abbreviations: *Q* Question

Q 1: Is the hypothesis/aim/objective of the study clearly described?; Q 2: Are the main outcomes to be measured clearly described in the introduction or methods?; Q 3: Are the characteristics of the subjects included in the study clearly described?; Q 4: Are the task procedures clearly described?; Q 6: Are the main findings of the study clearly described?; Q 11: Were the subjects asked to participate in the study representative of the entire population from which they were recruited?; Q 18: Were the statistical tests used to assess the main outcomes appropriate?; Q 20: Were the main outcome measures used accurate (valid and reliable)?; Q 27: Did the study have significant power to detect a clinically important effect where the probability value for a difference being due to chance is less than 5%?

### Demographic characteristics

Demographic characteristics of the subjects are presented in Table 2.

**Table 2:** Demographic characteristics

References	Samples	Gender	Age	Height	Weight
Rozzi et al. [34]	University athletes football, basketball	M = 17	20.4 ± 1.7	181.5 ± 7.2	80.3 ± 10.3
		F = 17	18.9 ± 0.9	168.5 ± 4.9	65.6 ± 8.3
Garrison et al. [40]	Football players	M = 8	19.3 ± 1.5	182.9 ± 2.4	77.1 ± 6.9
		F = 8	22.1 ± 2.4	168.6 ± 6.8	61.8 ± 3.2
Zazulak et al. [42]	Football players	M = 9	N	180.6	76.5
		F = 13	N	168.1	64.6
Carcia and Martin [39]	Recreational athletes	M = 10	22.82 ± 2.52	178.44 ± 6.45	178.44 ± 12.09
		F = 10	25.56 ± 2.49	169.99 ± 7.02	63.44 ± 7.02
Nagano et al. [41]	Basketball and tennis players	M = 18	19.8 ± 4.6	177 ± 40	68.7 ± 16.2
		F = 19	19.4 ± 0.9	166 ± 10	60 ± 7.5
Ebben et al. [32]	University Athletes	M = 12	21 ± 1.2	N	81.61 ± 13.3
		F = 12	19.91 ± 0.9	N	64.36 ± 6.14
de Britto et al. [31]	Recreational athletes	M = 10	28.9 ± 4	182 ± 7	81 ± 11
		F = 8	28.4 ± 6	167 ± 6	59 ± 6
Ogasawara et al. [33]	Recreational athletes	M = 17	24.8 ± 4.3	172.7 ± 6.5	70.9 ± 9.1
		F = 17	23 ± 1	161.3 ± 4.2	53.1 ± 7.4

*F* female, *M* male, *N* not reported

### Synthesis of results

Two authors (ZH and MSA) completed the analysis using both Microsoft Excel and Review Manager Version 5.4 (The Cochrane Collaboration, Denmark). Fixed and random-effects meta-analysis was used to analyze the results, with the I^2^ statistic being used to assess study heterogeneity. Whenever I^2^ was greater than 50%, indicting the existence it was e of high heterogeneity, the random effect size was used [44].

## Results

**Fig. 1:**
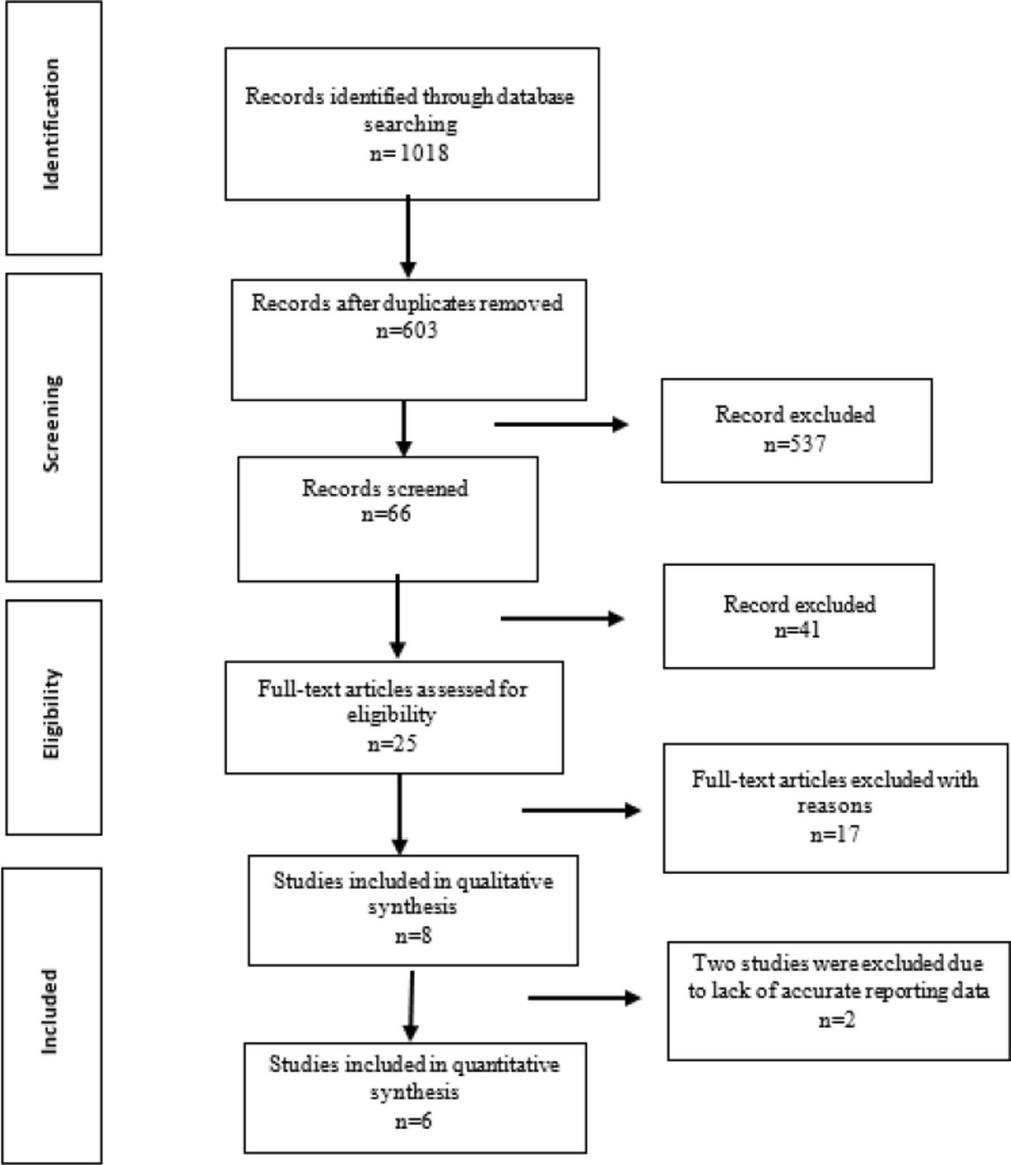
PRISMA Flow diagram showing the flow of information in the procedure of including studies in systematic review and meta-analysis

Of 1018 articles found in the databases based on the keywords, 415 duplicate articles were removed. After the screening of titles and abstracts, 537 articles were excluded. Thus, sixty-six full-text papers were reviewed and forty-one articles were excluded from the study according to the inclusion and exclusion criteria. Seventeen articles were excluded from the study because they did not provide the type of task or muscle information we were looking for; for example, they just provided information about female athletes or did not directly compare men and women [45, 46], or the task presented in them differed from our standard [47]. Finally, eight articles were reviewed qualitatively, and six articles were reviewed quantitatively (Fig. 1).

EMG data were presented as a percentage of the maximum voluntary isometric contraction,^1^ mean contractions,^2^ or normalized Root Mean Square (RMS). There were differences in the number of times the task was performed, the moment of measurement, and the height of the jump so that the height of the jump varied from 20 cm [31] to 60 cm [40] (Table 3).

**Table 3:** An overview of the characteristics of the main studies

Muscle	References	Task	Height cm	Measurement	Time measurement	Men Mean ± SD	Women Mean ± SD
RF	Garrison et al. [40]	SLL	60	Mean RMS	First contact	8.55 ± 5.65	7.81 ± 3.48
	Zazulak et al. [42]	SLL	30.5	Max MVC	200 ms BFC	18.7 ± 8.2	*33.6 ± 18.5
				Mean MVC		9.4 ± 5.2	13.9 ± 9.1
				Max MVC	250 ms AFC	45.1 ± 25.0	66.2 ± 31.9
				Mean MVC		25.8 ± 14.9	39.6 ± 19.6
	Nagano et al. [41]	SLL	30	MVC	50 ms BFC	a	a
					50 ms AFC	a	a
	Debritto et al. [31]	BDJ	20	NEMG	10 to 100 ms BFC	a	a
			40			a	a
	Ebben et al. [32]	BDJ	N MVJ	Max MVC	BFC	26.7 ± 21.92	19.74 ± 7.40
				Mean MVC	AFC	64.71 ± 46.32	49.81 ± 21.46
				Max MVC	BFC	69.44 ± 35.19	57.67 ± 8.09
				Mean MVC	AFC	224.17 ± 41.63	236.97 ± 23.13
VM	Rozzi et al. [34]	SLL	25.4	Mean onset time	Onset time	30.60 ± 51.98	39.20 ± 56.66
				Max MVC	Amplitude	290.87 ± 173.62	361.65 ± 255.49
	Ogasawara et al. [33]	SLL	32	Mean PV MVC	IFC	84.2 ± 22.8	142.5 ± 42.3
				Mean PT MVC		115 ± 38.4	93.6 ± 38.6
	Debritto et al. [31]	BDJ	20	NEMG	10 to 100 ms BFC	a	a
			40			a	a
	Ebben et al. [32]	BDJ	N MVJ	Max MVC	BFC	40.07 ± 30.64	37.99 ± 30.26
				Mean MVC	AFC	80.50 ± 39.56	85.09 ± 36.94
				Max MVC	BFC	*64.8 ± 12.20	*52.30 ± 9.77
				Mean MVC	AFC	223.36 ± 30.64	236.80 ± 18.4
VL	Rozzi et al. [34]	SLL	25.4	Mean onset time	Onset time	52.94 ± 70.52	40.51 ± 28.21
				Max MVC	Amplitude	298 ± 231.27	315.81 ± 162.5
	Garrison [40]	SLL	60	Mean RMS	First contact	14.88 ± 6.93	9.69 ± 3.58
	Ogasawara et al. [33]	SLL	32	Mean PV MVC	IFC	76.4 ± 17.7	115.6 ± 23.6
				Mean PT MVC		105.3 ± 46.1	97.8 ± 42
	Ebben et al. [32]	BDJ	N MVJ	Max MVC	BFC	30.49 ± 13.20	24.97 ± 16.43
				Mean MVC	AFC	97.61 ± 61.17	89.73 ± 51.47
				Max MVC	BFC	*62.08 ± 16.28	*46.95 ± 10.1
				Mean MVC	AFC	225.7 ± 37.28	236.08 ± 29.69
BF	Nagano et al. [41]	SLL	30	MVC	50 ms BFC	a	a
					50 ms AFC	a	a
	Garrison et al. [40]	SLL	60	Mean RMS	First contact	8.97 ± 6.76	6.13 ± 3.15
	Rozzi et al. [34]	SLL	25.4	Mean onset time	Onset time	217.63 ± 108.95	187.01 ± 133.19
				Max MVC	Amplitude	84.84 ± 43.67	156 ± 72.59
	Ogasawara et al. [33]	SLL	32	Mean PV MVC	IFC	30.8 ± 17.9	31.6 ± 17.9
				Mean PT MVC		8.8 ± 45.1	32.8 ± 28
	Debritto et al. [31]	BDJ	20	NEMG	10 to 100 ms BFC	a	a
			40			a	a
	Ebben et al. [32]	BDJ	N MVJ	Max MVC	BFC	26.24 ± 21.40	16.68 ± 13.1
				Mean MVC	AFC	*41.23 ± 43.32	*16.67 ± 16.11
				Max MVC	BFC	79.97 ± 23.91	81.74 ± 18.95
				Mean MVC	AFC	204.09 ± 39.94	215.14 ± 29.32
SM	Nagano et al. [41]	SLL	30	MVC	50 ms BFC	a	a
					50 ms AFC	a	a
	Rozzi et al. [34]	SLL	25.4	Mean onset time	Onset time	182.44 ± 91.88	175.57 ± 108.56
				Max MVC	Amplitude	134.20 ± 66.33	163.49 ± 84.45
	Ogasawara et al. [33]	SLL	32	Mean PV MVC	IFC	30.2 ± 17.4	27.7 ± 7.7
				Mean PT MVC		64.2 ± 41.3	12.9 ± 48.7
	Debritto et al. [31]	BDJ	20	NEMG	10 to 100 ms BFC	*a	*a
			40			a	a
	Ebben et al. [32]	BDJ	N MVJ	Max MVC	BFC	39.51 ± 45.93	36.32 ± 47.30
				Mean MVC	AFC	27.24 ± 27.12	23.71 ± 27.94
				Max MVC	BFC	103.56 ± 96.22	80.72 ± 22.19
				Mean MVC	AFC	2008.89 ± 68.15	2009.94 ± 26.67
MG	Rozzi et al. [34]	SLL	25.4	Mean onset time	Onset time	289.09 ± 177.96	241.1 ± 141.57
				Max MVC	Amplitude	134.13 ± 74.70	225.86 ± 223.35
LG	Rozzi et al. [34]	SLL	25.4	Mean onset time	Onset time	44.19 ± 98.58	193.90 ± 155.33
				Max MVC	Amplitude	161.45 ± 73.82	131.72 ± 64.90
GMax	Zazulak et al. [42]	SLL	30.5	Max MVC	200 ms BFC	47.4 ± 31.6	31.1 ± 18.2
				Mean MVC		16.3 ± 10.5	12.3 ± 7.6
				Max MVC	250 ms AFC	*98.0 ± 33.4	*69.5 ± 30.2
				Mean MVC		*53.9 ± 18.0	*37.5 ± 15.6
GMed	Garrison et al. [40]	SLL	60	Mean RMS	First contact	7.4 ± 4/85	3.84 ± 2.37
	Carcia and Martin [39]	BDJ	30	Max MVC	BFC	36.1 ± 16.17	51.0 ± 50.1
				Mean MVC		18.1 ± 6.2	21.3 ± 11.3
				Max MVC	AFC	111.1 ± 45.3	121.1 ± 62.1
				Mean MVC		61.6 ± 22.3	72.6 ± 42
	Zazulak et al. [42]	SLL	30.5	Max MVC	200 ms BFC	48.4 ± 27	39.6 ± 16.5
				Mean MVC		26 ± 17.8	20.8 ± 10.4
				Max MVC	250 ms AFC	79.3 ± 30.4	69.2 ± 28.2
				Mean MVC		43.2 ± 13.7	39.9 ± 18.5
	Ogasawara et al. [33]	SLL	32	Mean PV MVC	IFC	40.3 ± 13.4	46.4 ± 23.4
				Mean PT MVC		69.9 ± 49.5	56.8 ± 55.3

^a^Shows variables without numeric data, *Shows a significant difference between men and women (p < 0.05)

Abbreviations: *MVC* maximum voluntary contraction, *RMS* root mean square, *N EMG* Normalized myoelectric activity, *SLL* single-leg landing, *BDJ* bilateral drop jumps, *BFC* before foot contact, *AFC* after foot contact, *N MVJ* normalized with MVJ, *Mean PT MVC* mean peak time of % MVC, *Mean PV MVC* mean peak value of % MVC, *IFC* initial foot contact, Onset Time = Time (in milliseconds) from ground contact when landing a jump until the onset of muscle contraction, Amplitude = Peak amplitude (in millivolts) of the first contraction subsequent to landing a jump

Numerous studies have examined muscle activation in the feedforward and feedback stages (before and after foot contact with the ground) during the jump–landing task to determine gender differences related to ACL injuries. The jump–landing task included two-legged landings from heights of 20 and 40 cm [31], 30 cm [39], and jump–landings that were normalized for the subjects' height [39].

The subjects of the study made single-legged landings from a height of 25.4 cm [34, 44], 30 cm [41], 32 cm [33], 30.5 cm [42], 60 cm [40]. Generally, these studies provide a basis for understanding gender differences in activating the functional muscles of the ankle, knee, and pelvis during jumping and landing.

Early ground contact was the most common time for the measurements of EMG. The BF (6 studies) [31, 34] and the SM muscles (5 studies) 31[-34, 41] were measured more than other muscles. Various methods were used to measure muscle activation; in two studies [32, 34], the time of muscle activation was reported in relation to the initial contact of the foot with the ground, while in all eight studies [31–34, 39–43], maximum or average muscle activity was reported at specific intervals before or after ground contact. Sex differences in RF muscle activation were seen in one study [42] out of 5 studies [31, 32, 40–42]. However, Ebben [32] showed that the RF muscle was used in women significantly earlier.

Out of the 6 studies, Only Ebben [32] reported significant sex differences in external hamstring muscle activation (BF) [39–44]. In Ebben’s study, men showed more activity before and after ground contact than women. Four studies examined the mean and maximum activity of the VM muscle [39, 40, 43, 44]. Meanwhile, Ebben's study examined activity timing, showing that the VM muscle was activated earlier in women [32].

GMed muscle activation was measured in 4 studies [33, 39, 40, 42], and no significant differences were reported in GMed activation between the sexes. Zazolak [42] studied the mean and maximum activity of the GMax muscle, the results of which showed that the mean and maximum muscle activity after foot contact with the ground was higher in men than women [42]. But before contact with the ground, there was no significant difference between the two sexes.

### Muscle activity of biceps femoris, vastus medialis and rectus femoris

**Fig. 2:**
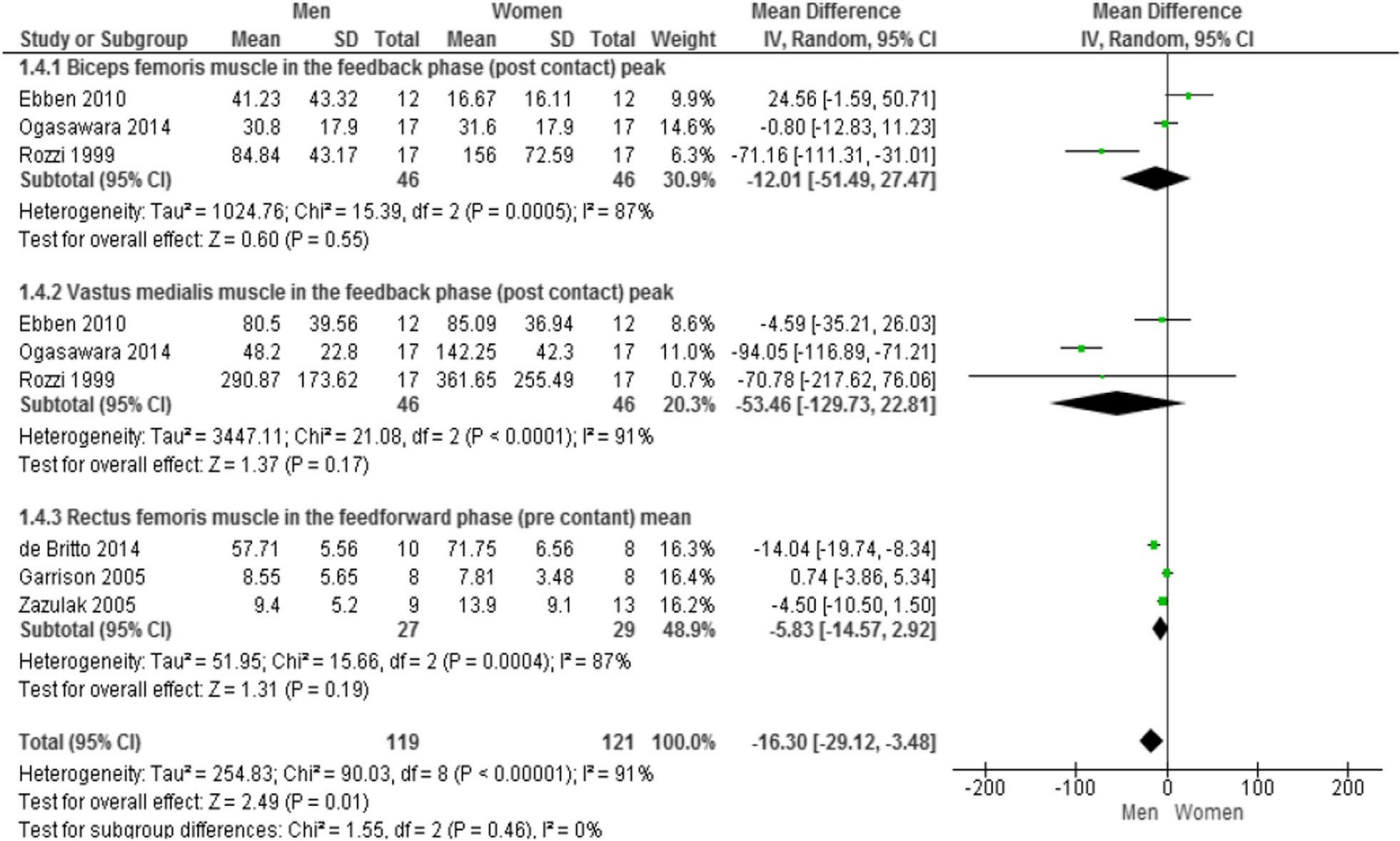
Forest plot of Comparison of biceps femoris, vastus medialis, and rectus femoris muscle activities between men and women in the feedback phase. Abbreviations: *IV* inverse Variance, *CI* confidence interval, *SD* standard deviation

Three studies compared the muscle activity of the BF and VM between men and women in the feedback phase [32–34]. The pooled data indicated that there was no significant difference between male and female muscle activity in BF (MD = −12.01; 95% CI − 51.49 to 27.47; p = 0.55; I^2^ = 87%) and VM muscles (MD = −53.46; 95% CI − 129.73 to 22.81; p = 0.17; I^2^ = 91%). In addition, three studies evaluated the muscle activity of the RF in the feedforward phase [31, 40, 42]. The pooled data indicated that there was no significant difference between male and female muscle activity in the RF muscle (MD = −5.83; 95% CI − 14.57 to 2.92; p = 0.19; I^2^ = 87%) (Fig. 2).

### Muscle activity of semimembranosus, vastus lateralis, and gluteus medius

**Fig. 3:**
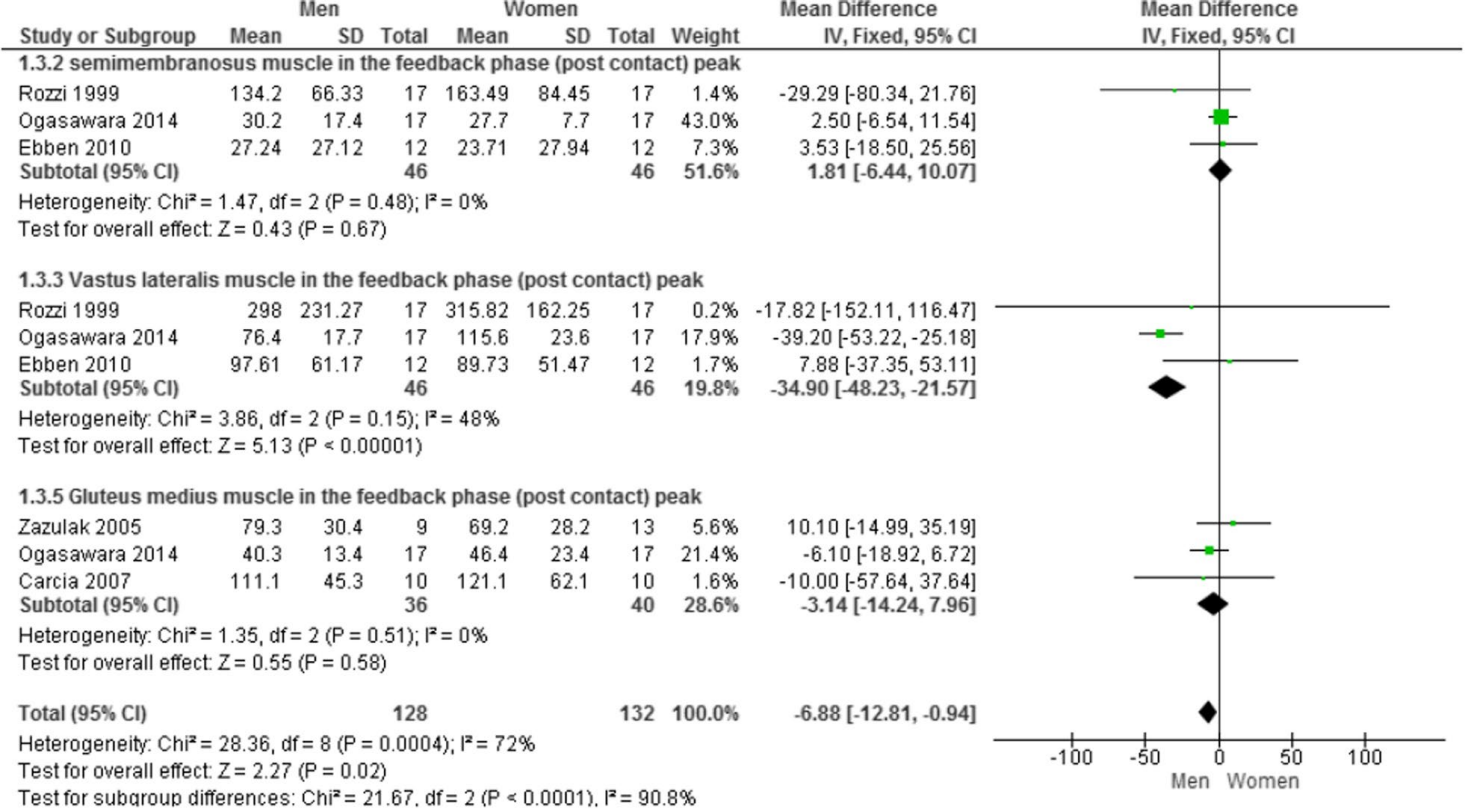
Forest plot of comparison of the SM, VL, and GMed muscle activity between men and women in the feedback phase. Abbreviations: *IV* inverse Variance, *CI* confidence interval, *SD* standard deviation

The pooled data indicated that there was no significant difference between male and female muscle activity in the SM muscle (MD = 1.81; 95% CI − 6.44 to 10.07; p = 0.67; I^2^ = 0%) [32–34], in the GMed (MD = −3.14; 95% CI − 14.24 to 7.96; p = 0.58; I^2^ = 0%) [33, 39, 42], and in the VL muscle (MD = −34.90; 95% CI − 48.23 to − 21.57; p < 0.00001; I^2^ = 48%) [32–34] (Fig. 3).

### Timing of semimembranosus and biceps femoris muscle activities

**Fig. 4:**
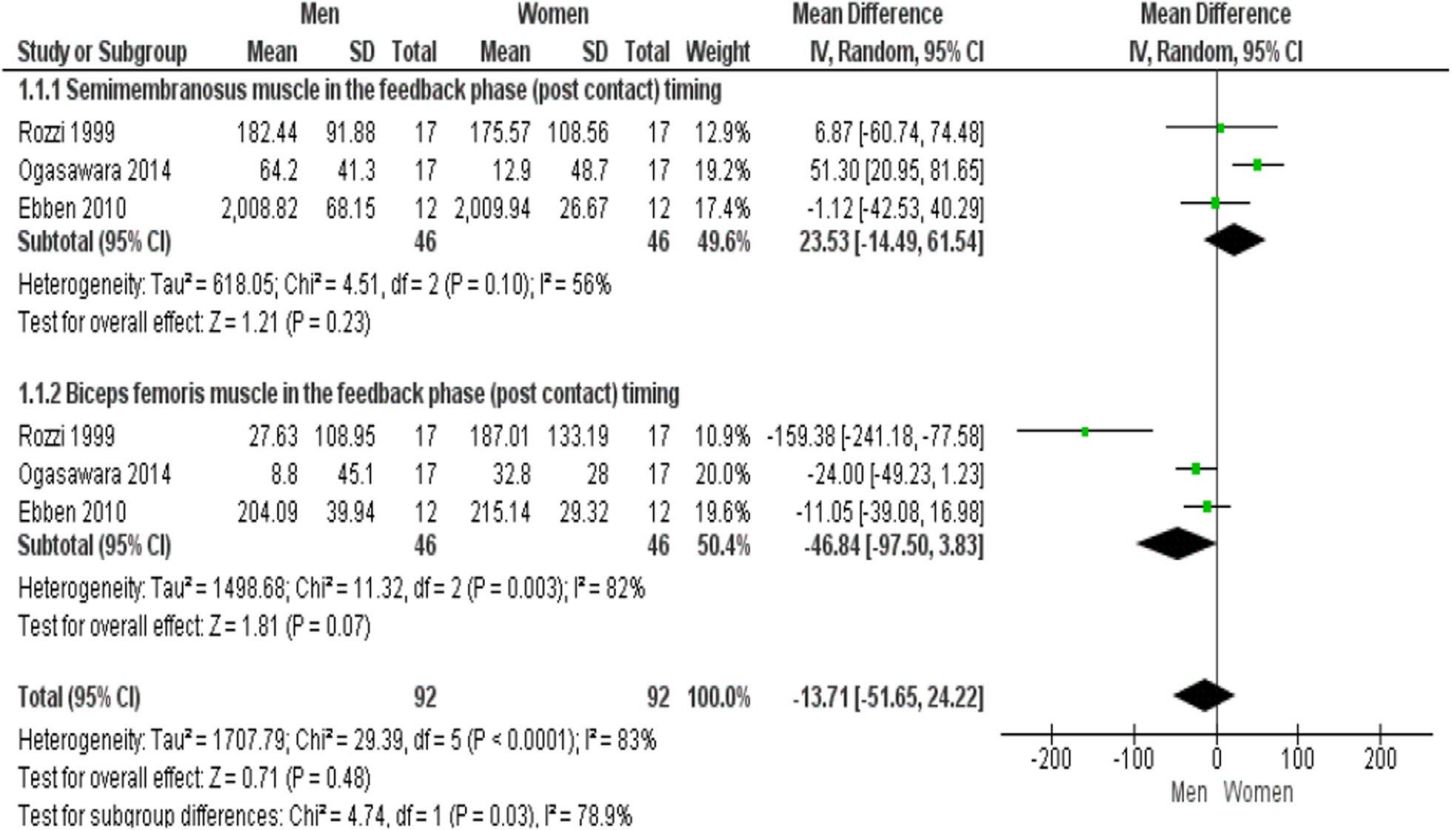
Forest plot of comparison of SM and BF timing between men and women. Abbreviations: *IV* inverse variance, *CI* confidence interval, *SD* standard deviation

Three studies evaluated the timing of SM and BF muscle activities between men and women in the feedback phase [32–34]. The results of the meta-analysis showed there was no significant difference between the sexes in the timing of SM muscle (MD = 23.53; 95% CI − 14.49 to 61.54; p = 0.23; I^2^ = 56%) and BF muscle (MD = −46.84; 95% CI − 97.50 to 3.83; p = 0.07; I^2^ = 82%) (Fig. 4).

## Discussion

The aim of this systematic review and meta-analysis was to compare the electromyographic activity of lower limb muscles in GMed, GMax, RF, VM, VL, BF, SM, MG and LG between men and women in the jump–landing task. Timing and muscle activity of eight articles from 1018 studies were comprehensively reviewed in the feedforward and feedback stages. Synthesised data showed no significant differences in muscle activity of BF, VM, SM, GMed and RF in the feedback and feedforward phases between the two sexes. However, there was a significant difference between the two sexes in the activity of the VL muscle in feedback phase. No significant difference observed between the two sexes in the timing of SM and BF muscles activity.

The mechanism of ACL injuries differs between genders and it has shown that ACL injuries is more common among women than men [8]. Many studies have attempted to explore the exact reason for this gender-based difference in ACL injuries. Although the exact reason still remains unclear, it seems that there are various factors such as sex hormones, anatomical differences, and neuromuscular control that lead to the higher ACL injuries in women [31–34, 39–42, 48]. Neuromuscular control defects are defined as improper patterns of activation, low muscle strength and power in the trunk and lower limbs, which lead to increased loads on the knee joint during sport activities [17, 49]. Differences between genders in neuromuscular activation patterns were reported to contribute to ACL injury [50]. Female athletes have been found to have various patterns of movement and muscle activation [51, 52]. Females increase the load on the ACL while jumping, because of increased quadriceps activation and reduced hip and knee flexion [53]. A study carried out by Anderson et al. revealed that the lack of rigidity and strength of quadriceps and hamstrings in females with anatomically smaller ACLs make them susceptible to injury [51]. Female athletes also displayed a greater laxity in the knee than their male counterparts and compared to men, women were found to be less effective in stiffening their knees [54, 55]. This excessive joint laxity appears to contribute to decreased joint proprioception, potentially increasing the risk of ACL injury [34]. The maximum contraction of the knee muscles significantly reduced anterior tibial translation in men and women comparing relaxed to contracted states [50]. Thus, neuromuscular utilization patterns and contraction rate, muscle utilization sequence and lower limb muscle feedback, and feedforward activity play an essential role in providing knee joint stability and preventing injury [56].

Five researchers have studied the amount of the RF muscle activity during jumping from different heights [31, 32, 40–42]. It has been shown that with the increase of height, the activity of the RF increases [45]. In two studies, the results showed that the feedforward activity of the RF muscle was significantly higher in women than in men [41, 42]. While in the other three studies, there was no difference between the two sexes in the feedforward or feedback activity of the RF muscle [31, 32, 40]. The difference in the studies can related to the workload caused by the type of task. Given that some studies have shown that women can jump 75.2% of men [57], at equal heights women may experience more intensity in muscle activity. Therefore, in two-legged jumping activities from a height of 40 cm or in single-legged jumping from a height of 30.5 and 30 cm, women showed more feedforward activity. But, when the height was proportional to the maximum vertical jump of individuals did not observe any difference between the two sexes [32]. Debrito et al. [31] from a height of 20 cm and Garrison et al. [40] from a height of 60 cm did not observe a difference between the two sexes. From what has been said, it can be concluded that if the intensity of activity is normalized to people's ability, there may be no significant difference between the two sexes regarding the feedforward and feedback activity of the RF muscle.

Our results of meta-analysis showed that there was no significant difference between the mean muscle activity of vastus medialis but there was a significant difference between the mean muscle activity of vastus lateralis in both sexes [32–34]. The VM and VL muscles activity were examined in three studies in the feedback phase. Ebben et al. [32] and Rozzi et al. [34] did not observe a significant difference, but Ogasawara et al. [33] observed a significant difference between the average muscle activity between men and women. However, Ebben et al. [32] reported faster activation of the VL muscle in the feedforward stage in women than men. The meta-analysis also showed a significant difference in the maximum activity of the VL muscle in the feedback phase between the two sexes, but there was no significant difference in the VM muscle [32–34]. In these three studies, the single leg jumping-landing task has been examined, and as mentioned earlier, the ability of women is 75.2% compared to men [57]. The different results of study of Ogasawara et al. [33] can probably be attributed to the high intensity of the task experienced by women. In the study of Rozzi et al. [34] both sexes jumped from the same height of 25.4 cm, which was a relatively light task for both groups. In the study of Ebben et al. [32] the jump height was adjusted to the abilities of the two sexes, so no difference was observed. But in Ogasawara's research, the jump height for both sexes was 32 cm, which was a task with high intensity for women [33]. On the other hand, various studies in the past have shown that women generally have more quadriceps muscle activity than men, and also that women’s knees tend to be more valgus [58, 59]. During landing, women showed a knee valgus posture at the time of ACL injury [12]. In general, if the jump height is commensurate with the ability of women, they learn to control the muscles activity of the vastus medialis and vastus lateralis in order to prevent knee valgus posture and thus prevent ACL injury.

Six studies examined the BF muscle activity in male and female athletes in the feedforward phase and did not observe a significant difference between them [31–34, 40, 41]. Two researchers also examined the average muscle activity of BF in the feedback stage and did not observe a significant difference [40, 41]. Of the above six studies, three were included in the meta-analysis [32–34]. There were no significant differences in maximum muscle activity and timing of BF muscle activity in two studies [32, 33] and a significant difference in one study [34]. In general, the pooled data showed no significant difference between the two sexes in maximum activity and timig of BF in the feedback phase [32–34]. Given that the samples of Rozzi et al. [34] were professional basketball and futsal athletes and that their jump height was low (25.4 cm), females have probably learned to activate their hamstring muscle more. However, the research samples of Ebben et al. [32] were people who were not professional athletes and the jump height was normalized with the ability of the subjects. Another factor to consider was the measurement time [32]. Ogasawara et al. [33] and Rozzi et al. [34] considered the first contraction at the moment of contact with the ground as feedback activity. While Ebben et al. [32] considered the maximum electromyographic activity between the first foot contact with the ground to 125 ms later.

Five studies compared the activity of the SM muscle after foot contact with the ground in men and women in the jump–landing task and found no significant difference [31, 33, 34, 39, 41, 60]. However, Debrito et al. [31] assessed two-leg jump–landing from the heights of 20 and 40 cm in the feedforward stage and observed that the activity of SM muscle in women before foot contact with the ground is significantly higher than men. This difference in results may be due to differences in task performance because single-leg and two-leg landing are different in intensity and muscles activation mechanism. However, in terms of activation time, no difference was reported between the two sexes in the feedforward and feedback stages [32, 34]. Of the above five studies, three were included in the meta-analysis [32–34]. There were no significant differences in maximum muscle activity of SM muscle activity in three studies [32–34]. Of the above five studies, three were included in the meta-analysis [32–34]. There were no significant differences in timing of SM muscle activity in two studies [32, 34] and a significant difference in one study [33]. In general, the pooled data of meta-analysis did not showed any significant difference between the two sexes in maximum activity and timig of SM muscle in the feedback phase [32–34]. In addition to sex differences in the magnitude of activation, differences in the timing of hamstring muscles (SM and BF) and quadriceps activation have not been illustrated in some researches [34, 61], while other researchers have found differences with male producing delayed SM onset at foot contact, compared with female, which was idea to be a defensive mechanism permitting most hamstring activation to correspond with the timing of anterior tibial shear [48, 62].

Based on a systematic review, Garrison et al. [40], Carcia and Martin [39], Zazulak et al. [42], and Ogasawara et al. [33] examined GMed muscle activity as feedforward and feedback at different jump heights. They did not observe any significant differences in the amount and timing of muscle activity [33, 39, 40, 42]. The GMax muscles and external rotators of the hip appear to play a more critical role than the GMed in controlling the tibiofemoral joint. The GMax is considered to control the internal hip rotation and flexion [63]. The results of a meta-analysis of GMed muscle activity in the feedback phase showed that there was no significant difference between the two sexes after foot contact with the ground [33, 39, 40, 42]. GMed muscle activity was similar between sex after ground contact when subjects performed a jump–landing. Women subjects exhibited increased variability in GMed EMG during a jump–landing when compared to men [39]. Additional studies were necessary before firm conclusions regarding the effect of sex on jumping and landing tasks can be made [39].

To our knowledge this is the first study to systematically examine gender differences in lower limb muscle activity during the jump–landing tasks. However, the study has several limitations worth noting. One is that only articles published in English were reviewed. Another one is that only the jump–landing task was investigated. So, these results cannot be generalized to other tasks such as cutting tasks.

## Conclusion

This systematic review and meta-analysis showed that in most lower limb muscles, there was no significant difference between muscle activity and muscle contraction timing in both sexes before and after foot contact. A significant difference between the two sexes showed just in the vastus lateralis muscle. Overall, it can be concluded that the reason for the greater susceptibility of ACL injuries in women than men is maybe related to other factors such as biomechanical and hormonal. Also, it was found that the level of physical fitness can affect the pattern of muscle activation. Therefore, if the intensity of the jump–landing task is commensurate with the ability of men and women, the two sexes exhibit similar patterns of muscle activation before and during the jump–landing task. Additional good quality research in this regard is required to strengthen these conclusions.

## Data Availability

All data generated or analysed during this study are included in this published article.

## Footnotes

1 Peak % MVC.

2 Mean % MVC.
